# Dutch Guideline on Knee Arthroscopy Part 1, the meniscus: a multidisciplinary review by the Dutch Orthopaedic Association

**DOI:** 10.1080/17453674.2020.1850086

**Published:** 2020-11-24

**Authors:** Ewoud R A Van Arkel, Sander Koëter, Paul C Rijk, Tony G Van Tienen, Patrice W J Vincken, Michiel J M Segers, Bert Van Essen, Nicky Van Melick, Bernardine H Stegeman

**Affiliations:** a FocusKliniek Orthopedie, Haaglanden Medisch Centrum , Den Haag ;; b Department of Orthopedic Surgery, Canisius Wilhelmina Ziekenhuis , Nijmegen ;; c Department of Orthopedic Surgery, Medisch Centrum Leeuwarden , Leeuwarden ;; d Department of Orthopedic Surgery, Radbout UMC , Nijmegen ;; e Department of Radiology, Alrijne Hospital , Leiderdorp ;; f Department of Surgery, Sint Antonius Ziekenhuis , Utrecht ;; g Department of Sportsmedicine, Maxima Medisch Centrum , Veldhoven ;; h Knee Expert Center , Eindhoven ;; i Kennisinstituut van de Federatie Medisch Specialisten , Utrecht , The Netherlands

## Abstract

Background and purpose — A guideline committee of medical specialists and a physiotherapist was formed on the initiative of the Dutch Orthopedic Association (NOV) to update the guideline Arthroscopy of the Knee: Indications and Treatment 2010. This next guideline was developed between June 2017 and December 2019. In this Part 1 we focus on the meniscus, in Part 2 on all other aspects of knee arthroscopy.

Methods — The guideline was developed in accordance with the criteria of the AGREE instrument (AGREE II: Appraisal of Guidelines for Research and Evaluation II) with support of a professional methodologist from the Dutch Knowledge Institute of Medical Specialists. The scientific literature was searched and systematically analyzed. Conclusions and recommendations were formulated according to the Grading of Recommendations Assessment, Development, and Evaluation (GRADE) method. Recommendations were developed considering the balance of benefits and harms, the type and quality of evidence, the values and preferences of the people involved, and the costs.

Here in Part 1 we focus on the diagnosis of meniscal pathology, treatment of meniscal injuries, and persistent pain after meniscal repair and after treatment. In this guideline, the importance of nonoperative treatment of degenerative meniscal tears, the potential for spontaneous healing of traumatic meniscal tears, and encouragement to repair rather than resect meniscal tears when possible are highlighted.

The guideline is published online, in Dutch, and is available from the Dutch Guideline Database (https://richtlijnendatabase.nl/?query=Artroscopie+van+de+knie+&specialism=)

The Dutch Orthopedic Association (NOV) has a long tradition of development of practical clinical guidelines with the use of the GRADE method. This is a systematic and transparent approach to collecting and grading of the available evidence and to weighing the evidence together with complementary arguments, so-called considerations, relevant to the clinical question—including patient values and preferences, and resource use (cost, organization of care issue). It is a dynamic tool in which a particular module can be altered if there are new insights (Besselaar et al. 2017).

The guideline Knee Arthroscopy describes the indication, diagnosis, and treatment for knee arthroscopy. For better readability we have divided the guideline into 2 parts. In Part 1 we focus on the meniscus. In Part 2 we discuss all other aspects of arthroscopic knee surgery (Koëter et al. 2020). We do not address knee arthroscopy in children, with specific diagnoses such as discoid meniscus and osteochondritis dissecans.

The implementation of the guideline Arthroscopy of the Knee: Indications and Treatment 2010 contributed to a decrease in incidence of meniscal procedures in all age groups in the Netherlands from 2005 to 2014, with a more pronounced decrease in the younger patients (Rongen et al. 2018). Due to “game changing” studies on the meniscus and further technical developments in the field of arthroscopic treatment of knee complaints and developments of the technique of MRI, it became necessary to revise the 2010 guideline Arthroscopy of the Knee.

5 clinical questions on the meniscus were formulated by a steering group of the Dutch Orthopedic Association (see Guideline recommendations below).

This guideline aims to provide a uniform policy for the care of patients with knee injuries that could possibly be treated with an arthroscopic procedure. It is written for orthopedic surgeons, sport medicine specialists, physiotherapists, radiologists, and trauma surgeons who are involved in the care of patients with (acute) knee injuries. In addition, this guideline is intended to inform healthcare providers who are also involved in the care of these patients, including pediatricians, rehabilitation doctors, general practitioners, physician assistants, and nurse practitioners.

## Funding and potential conflicts of interest

The guideline development was financially supported by the Dutch Orthopaedic Association (NOV), using governmental funding from the Quality Foundation of the Dutch Association of Medical Specialists in the Netherlands. The authors declare that there is no relevant conflict of interest.

## Method

### Guideline panel

In November 2016, a guideline panel, tasked with revising the guideline, was formed consisting of orthopedic surgeons, a radiologist, a trauma surgeon, a physiotherapist, and a sports medicine specialist. A methodologist from the Knowledge Institute of the Federation of Medical Specialists supported the guideline panel by ensuring proper design and systematic evidence-based development of the guideline using the GRADE methodology, to meet all the criteria of the AGREE instrument.

### Methodology and workflow

The guideline was developed in agreement with the criteria set by the advisory committee on guideline development of the Federation Medical Specialist in the Netherlands, which are based on the AGREE II instrument (Brouwers et al. 2010). The guideline was developed using an evidence-based approach endorsing the GRADE methodology, and meets all criteria of AGREE II. Grading of Recommendations Assessment, Development, and Evaluation (GRADE) is a systematic approach for synthesizing evidence and grading of recommendations offering transparency at each stage of the guideline development (Guyatt et al. 2011, Schünemann et al. 2014). The guideline development process involves a number of phases: a preparatory phase, a development phase, a commentary phase, and an authorization phase. After authorization, the guideline has to be disseminated and implemented. Furthermore, uptake and use must be evaluated. Finally, the guideline must be kept up-to date.

Each phase involves a number of practical steps (Schunemann et al. 2014). As a first step in the early preparatory phase, a broad forum discussion was held and all relevant stakeholders were consulted to define and prioritize key issues, which were extensively discussed in the guideline panel. The selected, high-priority issues were translated into carefully formulated clinical questions. These questions defined patient problems, intervention, comparison, and outcomes. Furthermore, the patient outcomes relevant to decision-making were prioritized and minimal clinically important differences were defined.

In the development phase, the literature was systematically searched using the databases MEDLINE and Embase. Selection of the relevant literature was based on predefined inclusion and exclusion criteria and was carried out by 1 member of the guideline panel (EvA) in collaboration with the methodologist (BS). For each of the clinical questions, the evidence was summarized by the guideline methodologist using the GRADE approach. A systematic review was performed for each of the relevant outcomes and the quality of evidence was assessed in 1 of 4 grades (high, moderate, low, very low) by analyzing limitations in study design or execution (risk of bias), inconsistency of results, indirectness of evidence, imprecision, and publication bias. The evidence synthesis was complemented by a guideline panel member considering any additional arguments relevant to the clinical question, including patient values, preferences, and resource use (costs, organization of care issues). Evidence synthesis, complementary arguments, and concept recommendations were extensively discussed in the guideline committee. Final recommendations were then formulated. The final recommendations are based on the balance between desirable and undesirable outcomes, the quality of the body of evidence across all relevant outcomes, values and preferences, and resource use. The strength of a recommendation reflects the extent to which the guideline panel was confident that desirable effects of the intervention would outweigh undesirable effects or vice versa, across the range of patients for whom the recommendation is intended. The strength of a recommendation is determined by weighing all relevant arguments together. This includes the weight of the body of evidence from the systematic literature analysis, and also the weight of all complementary arguments formulated, the so-called considerations. When using the GRADE approach, guideline panels must use judgment in integrating these arguments to make a strong or weak recommendation. Thus, although low quality of the body of evidence from the systematic literature analysis will generally result in a weak recommendation, it does not a priori exclude a strong recommendation, and weak recommendations may also result from high-quality evidence (Schunemann et al. 2014).

After reaching consensus from the guideline panel, the concept guideline was subjected to peer review by all the relevant stakeholders: the commentary phase. Amendments were made and agreed upon by the guideline panel, and the final text was presented to the Dutch Orthopedic Association (NOV), the Dutch Society for Radiology (NVvR), the Royal Dutch Society for Physical Therapy (KNGF), the Dutch Sports Medicine Association (VSG), and the Association of Surgeons of the Netherlands (NVvH) for authorization. In this authorization phase, additional amendments were made to the guideline text based on the outcome of a general assembly of the NOV. The guideline was finally approved and officially authorized by the NOV in March 2019.

**Guideline recommendations according to 5 clinical questions, for literature reviews, see Supplementary data**

### 1. What is the value of the different meniscus tests during physical examination?

#### Recommendation

•Do not perform arthroscopy based on 1 or more meniscus tests without additional information from history, physical examination, and any additional radiological examination.

### 2. What is the place of imaging techniques such as conventional radiographs, MRI, and ultrasound in the diagnostic process?

#### Recommendation

•Perform imaging, such as conventional radiographs and/or MRI, before performing an arthroscopy.

•Perform an MRI in addition to history and physical examination in patients younger than 50 years, unless there is a high a priori chance of intra-articular injury. In that case an arthroscopy without MRI is indicated (provided conventional radiograph is done). A high a priori chance is defined as: history of a traumatic moment, hydrops, and a locked knee.

•Do not routinely perform MRI in patients older than 50 years with knee complaints, but start with an AP and lateral radiograph of the knee, preferably with fixed flexion at 20°.

•Be cautious when applying ultrasound in the indication for arthroscopy due to insufficient visibility of (intra)osseous and intra-articular structures of the knee.

### 3. What is the additional value of CT and MRI arthrography compared with conventional MRI in patients with a meniscus repair after injury?

#### Recommendation

•Consider (direct) MR arthrography over conventional MRI as additional diagnostics for persistent complaints after an arthroscopic treatment of a meniscal injury.

### 4. Which meniscus injuries should be treated, when and how?

#### Recommendation

##### Acute meniscal injury

• Perform arthroscopy within 2 weeks of injury when a patient has a locked knee with the most likely cause of a ruptured meniscus.

• Always consider meniscal repair or follow a wait-and-see policy. Meniscal injury does not necessarily mean meniscectomy.

• Leave the peripheral rim of the meniscus intact.

• Always try to repair a meniscal tear in young patients if the tear is in the vascularized part of the meniscus. Here, a stable knee or an unstable knee that is stabilized within 6 weeks is indispensable.

##### Degenerative meniscus injury

•Start with nonoperative treatment in degenerative meniscus injury.

•Consider treating nonoperatively for at least 3 months in the event of a meniscal tear.

### 5. What is the added value of physiotherapy after arthroscopic meniscus surgery of the knee?

#### Recommendation

•Do not refer patients with an expected normal recovery to the physiotherapist after a meniscectomy.

•Discuss with the patient with a delayed recovery the expected effects of physical therapy.

## Discussion

The guideline is aimed at providing evidence-based advice to medical specialists and physiotherapists, in order to minimize unwarranted variation in treatment of meniscus injuries and to improve the therapeutic results.

The 1st question concerned the values of diagnostic meniscus tests. The execution of meniscus tests is part of the standard physical examination during consultation, in all patients with (non)traumatic knee complaints. Other findings during the physical examination, such as a locked knee, or swelling of the knee, may also support the suspicion of meniscal disease. A locked knee may be a reason to qualify a patient for arthroscopy without prior MRI, provided a conventional weightbearing knee radiograph has been taken to exclude other pathology. Analysis of the literature clearly shows that performing meniscus tests alone, separately or in combination is diagnostically insufficiently accurate (Goossens et al. 2015, Smith et al. 2015). In a combination of both a negative Thessaly and McMurray test, the presence of a meniscal injury is unlikely. Further caution with the use of meniscus tests is appropriate if there is concomitant ligamentous injury such as an anterior cruciate ligament rupture. This further decreases the reliability of the meniscus tests. Additional information from demographic factors, history, physical examination, and possibly additional examination (MRI) has to support the diagnosis before treatment decisions can be made.

**Figure:**
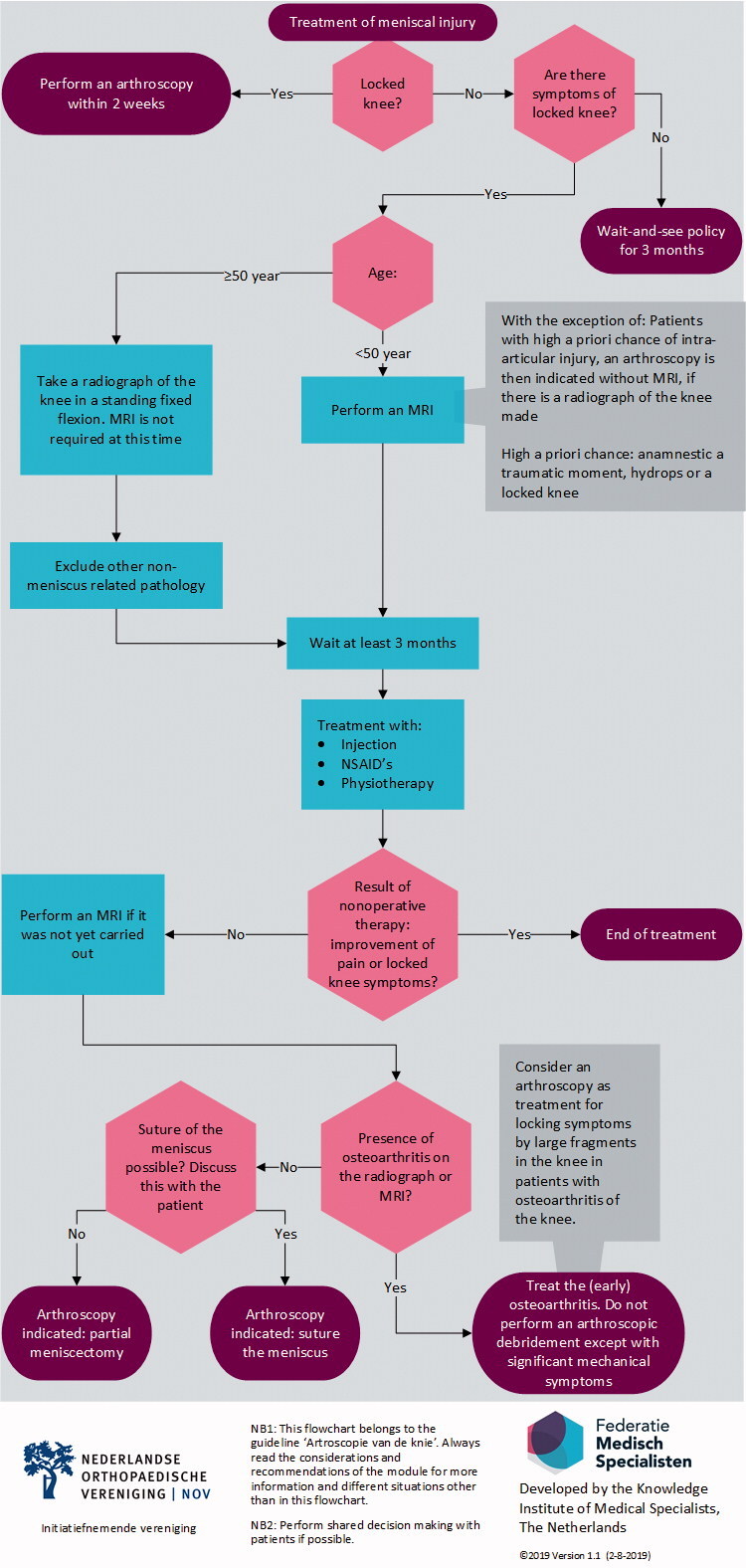


The 2nd question concerned the place and reliability of imaging of ultrasound compared with MRI techniques in the diagnostic process. Prior to arthroscopy osseous pathology (fracture, neoplasm, and infectious processes such as osteomyelitis) should be excluded by imaging. A general MRI scanning protocol, suitable for the assessment of menisci, ligaments, and cartilage, should consist of scans in different planes: sagittal, coronal, and, where relevant, axial. In the choice of 35 sequences at least a combination of short TE (T1- or PD-weighted images) and T2-weighted images with or without fat suppression should be used. Because there is a learning curve in reading of MR images, experience and training of the reader will increase the accuracy of MRI (White et al. 1997). Experience of the reader has more influence on accuracy of MRI than field strength of the system (Krampla et al. 2009). Therefore, reading of the MRI by a (musculoskeletal) radiologist, with adequate training and experience, is essential to achieve the highest accuracy.

A limited number of studies, compared with those on MRI, indicate that ultrasound of the knee can be accurate in the assessment of menisci and cruciate ligaments. Transducers used in the aforementioned studies ranged from 5 to 14 MHz. No information is available on the influence of the frequency of transducers on accuracy, but higher frequencies yield higher resolution images, probably increasing accuracy. Cartilage can be evaluated only to a minor extent. Bone and bone marrow cannot be assessed with ultrasound. This seriously limits the diagnostic yield of ultrasonography when compared with MRI.

The learning curve for performing musculoskeletal ultrasound is considerable, because not only the interpretation of the obtained images but also eye–hand coordination benefits greatly from training and experience. Therefore, ultrasonography is only a reliable diagnostic tool in the hands of an experienced musculoskeletal ultrasonographer.

Advocates of ultrasound point to the fact that availability of ultrasound machines is higher than that of MRI systems. This may be true, but the limiting factor will probably be the availability of adequately trained and experienced ultrasonographers. The disadvantage compared with MRI is that the ultrasonographer has to perform the examination in person to make an adequate report, whereas MRI can be reported by a radiologist independent of moment and location of examination. This facilitates planning. An MRI scan consists of multiple images of predefined thickness and interval in at least two orthogonal planes. This means every orthopedic surgeon or radiologist can identify and locate pathology in the knee based on information visible in these images. Because ultrasound is a dynamic examination and the number of obtained images, orientation, and quality of images is entirely operator-dependent; only the reporter/ultrasonographer can extract all the information from an examination. Others will have to rely on the report. This will diminish the added value of ultrasound in preoperative planning or in giving the patient insight into the pathology.

When arthroscopy is warranted, MRI can be used to exclude (oncologic) osseous pathology and radiography is not required. Ultrasound cannot fulfill that role because visualization of osseous structures is insufficient. Additional imaging (radiography) is required. This means that ultrasound cannot be a stand-alone diagnostic tool before arthroscopy. Another disadvantage of ultrasound is the inability to visualize bone marrow changes at all, or cartilage in a sufficient manner. Bone bruising or focal chondral lesions can mimic meniscal pathology and the bone bruise pattern can point to specific trauma mechanisms and associated pathology. And one could also assume the use of ultrasound in obese patients is limited. Compared with MRI, ultrasound therefore lacks in completeness.

In our opinion, MRI is the diagnostic imaging of choice in patients younger than 50 years and without history of locking or catching or extension deficit (history of trauma, effusion, and extension deficit) (Vincken et al. 2007). Ultrasound is not equivalent.

The 3rd question involved patients with persistent complaints after repair. MR arthrography may have additional value over conventional MRI, as there is evidence that MR arthrography has a higher accuracy for detecting unhealed or re-ruptured menisci. The cost of the study is likely to be higher due to the use of intra-articular contrast. There will also be a higher risk of complications due to the (minimally) invasive nature. No literature data is available showing which strategy (conventional MRI and possibly additional MR arthrography, MR arthrography alone, or direct second-look arthroscopy) is preferable from the point of view of cost-effectiveness or clinical outcome. CT arthrography is also believed to have higher accuracy for detecting unhealed or torn menisci than with conventional MRI. However, no article was included in our literature search confirming this. De Filippo et al. (2009) examined CT arthrography in patients with MRI contraindication, but studies comparing different imaging modalities are lacking. Availability, expertise, and local customs also play a role in these considerations.

The 4th question addressed the treatment of acute meniscus injury and degenerative meniscal lesions. Concerning acute meniscal injury, the working group is of the opinion that studies more than 8 years old, comparing total meniscectomy with partial meniscectomy, are no longer relevant, because standard care has changed in recent years. It is now preferable to repair a meniscus and if not possible then to perform an arthroscopic partial meniscectomy. Xu and Zhao (2015) undertook a meta-analysis of the comparison between meniscus repair and a meniscectomy with better outcomes for meniscus repair. There is currently insufficient scientific evidence to determine when and which meniscal lesions should be repaired or removed to obtain optimal outcomes in the short and longer term. However, it seems more prudent to have low-threshold suturing in younger patients with a lateral meniscal injury than to perform a partial meniscectomy because of the long-term results after lateral meniscectomy (Hulet et al. 2015).

The working group considers meniscus tears to be repairable when they are torn close to or separated from the knee joint capsule, or a longitudinal tear in the peripheral part the “so-called red-red zone” where the healing potential is best because of the vascularization, provided that the torn meniscus tissue is of good quality and the knee is stable. This usually concerns traumatic meniscal tears. Spontaneous healing of meniscal injuries has also been extensively described, both in combination with anterior cruciate ligament rupture and with isolated meniscal injuries. The working group is of the opinion that in the case of a peripheral longitudinal tear of the meniscus proven on MRI and no restriction of movement in the knee, a wait-and-see policy can be pursued. Due to the chance of spontaneous healing, overtreatment may result.

Recovery after meniscus repair takes longer than partial meniscectomy. No evidence-based post-treatment protocol is available, but it is generally advised to do partial weightbearing for 4 to 6 weeks and limit flexion to 90 degrees. Return to sport level is advised after 3 to 6 months. Postoperative rehabilitation should be discussed explicitly with a top athlete so that a well-considered decision can be made with regard to repair or partial meniscectomy. We recommend a different approach to medial vs. lateral meniscus tears. The younger the patient, the more aggressive the surgeon should be in repair of the lateral meniscus. And in the case of a bucket-handle tear in combination with an anterior cruciate ligament rupture, meniscus repair should be performed in combination with an ACL reconstruction.

The second part of the 4th question concerns degenerative menisci, as regards treatment of degenerative meniscal tears.

Most studies concerning degenerative meniscus injury used 3 months as the “short-term follow-up.” During the first 3 months after surgery and nonoperative treatment, a reduction in complaints was measured and the difference in effect of treatment between the two groups seemed minimal. This minimal difference continued up to 24 months. In case of nonobstructive meniscus complaints, conservative treatment is therefore preferable to surgery for the first 3 months after initiations of complaints. The working group has set the age limit for degenerative meniscal injuries to > 50 years, but this is open to debate. The reason for choosing 50 years was to stay in line with the knee osteoarthritis guideline and the associated radiographic diagnostics. Progressive insight shows that lowering the age of 45 years produces the same results and thus can also be considered. Perhaps in the future the age recommendation will further decrease to 35 years, but more research is needed.

The5th question addressed physical therapy. Physical therapy is an intervention that entails hardly any risks or complications. In the Netherlands, the first 20 physiotherapy treatments after surgery are reimbursed by the patient’s additional insurance. From the 21st treatment onward, reimbursement from the basic insurance applies up to 12 months after the meniscectomy. If the patient has less than 20 treatments in his additional insurance, he will therefore have to pay for physiotherapy treatments him- or herself.

Today’s society demands a return to work as soon as possible and physiotherapy may be able to contribute to this. In addition, it is often the wish of the patient to be able to quickly return to the old level, particularly when it comes to sports. In certain professional groups (top athletes, heavy physical work), counseling in postoperative recovery can therefore be useful, also to prevent secondary injuries. For example, it may be useful to monitor a top athlete more frequently in connection with a step-by-step build-up. This group of patients often ignore symptoms because they want to get back into competition quickly or because of pressure from the media, the coach, the team, or the athletes.

It is not only the type and treatment of meniscal injury that determines whether the patient should be referred to a physiotherapist, but more whether there is normal or (expected) delayed recovery. In the Royal Dutch Physical Therapists (KNGF) meniscectomy guideline two patient profiles are distinguished. Patient profile 1 concerns patients after partial meniscectomy who require little or no physical therapy because of expected normal recovery. These are usually the younger patients with an acute injury of the meniscus, with a blank history. Physiotherapy is desirable in this group only if there is comorbidity (such as an ACL rupture) or fear of movement.

Patient profile 2 are patients with a burdened history of previous knee surgeries, in whom the complaints have arisen after repeated (micro)trauma, causing multiple and recurrent ruptures in the meniscus. This may be a sign of nascent osteoarthritis, but the distinction between meniscus pathology and early stage degenerative knee disease may not be clear. These patients are at high risk of delayed recovery and physical therapy is indicated. Other signs of delayed recovery are insufficient increase in function (mobility, gait pattern) and insufficient increase or even decline in activities and participation. In that case, physical therapy can help improve mobility and gait recovery, and increase strength and neuromuscular control, which may also increase activity and participation. However, as discussed in this guideline, in most cases these patients are no longer even eligible for arthroscopy and therefore should be treated nonoperatively.

This guideline was composed for arthroscopic treatment of knee injuries. Here we present our recommendations concerning the meniscus. We should keep in mind that there is a continuum from a traumatic meniscal injury at a younger age to a degenerative meniscal lesion around midlife. We have changed our thoughts from performing partial meniscectomy in the case of meniscal lesions to first considering nonoperative treatment before meniscal repair. If surgery is indicated and meniscal repair is not possible partial meniscectomy should be considered. Spontaneous decrease in pain with meniscus lesions is possible. We should be aware that a degenerative meniscus lesion is one of the first signs of osteoarthritis of the knee.

## Supplementary data

Literature reviews are available as supplementary data in the online version of this article, http://dx.doi.org/10.1080/17453674.2020.1850086
